# 5-Fluorouracil Induced Takotsubo Cardiomyopathy Complicated by Left Ventricular Thrombosis

**DOI:** 10.7759/cureus.14049

**Published:** 2021-03-22

**Authors:** Dilpat Kumar, FNU Warsha, Aditya Mehta, Vishal Deepak, Wassim Jawad

**Affiliations:** 1 Internal Medicine, Western Michigan University, Kalamazoo, USA; 2 Internal Medicine, Interfaith Medical Center, Brooklyn, USA; 3 Internal Medicine, Western Michigan University Homer Stryker M.D. School of Medicine, Kalamazoo, USA; 4 Critical Care Medicine, West Virginia University School of Medicine, Kalamazoo, USA; 5 Electrophysiology, Spectrum Health Medical Group, Grand Rapids, USA

**Keywords:** takotsubo cardiomyopathy, broken heart syndrome, left ventricular thrombosis, apixaban and aspirin, 5-fluorouracil

## Abstract

A 42-year-old woman with a remote history of smoking and recently diagnosed anorectal cancer presented with typical anginal chest pain, dyspnea, palpitations, and hallucinations. She was started on continuous 5-flurouracil (5-FU) infusion five days before presentation. Her physical examination was significant for bilateral bibasilar crackles and tachycardia. Her bloodwork was significant for an increased troponin and brain natriuretic peptide (BNP). Electrocardiogram (EKG) showed sinus tachycardia with ST elevation in multiple contiguous leads, whereas transthoracic echocardiogram (TTE) showed estimated ejection fraction of 17% with severe global hypokinesis with apical akinesis and matted thrombus at the apex. Coronary angiogram showed 20% occlusion of the left anterior descending artery. She was diagnosed with 5-FU induced Takotsubo cardiomyopathy complicated by left ventricular (LV) thrombosis. 5-FU was discontinued, uridine triacetate was given as reversal agent. Aspirin and apixaban were started for three months for LV thrombosis. Her six-week TTE showed return of normal heart function with resolution of LV thrombosis.

## Introduction

The fluoropyrimidines, namely 5-fluorouracil (5-FU) and its oral prodrug, capecitabine, are antimetabolites, which are widely used for the treatment of solid tumors of glandular and squamous origin, such as those involving the head and neck, gastrointestinal tract, and bladder [1,2]. Its mechanism of action includes inhibition of thymidylate transferase and incorporation into deoxyribonucleic acid (DNA) and ribonucleic acid (RNA) [3,4]. 5-FU is the gold standard of adjuvant therapy with radiation in anorectal cancer. It is commonly associated with adverse reactions of diarrhea, mucositis, and myelosuppression, and, rarely, cardiotoxicity, with an incidence of 1.2% [2,3,5].

Takotsubo cardiomyopathy (TCM), also known as broken heart syndrome or stress-induced cardiomyopathy, is an acute cardiac syndrome characterized by transient left ventricular (LV) apical ballooning [6]. It is characterized by the abrupt onset of angina-like chest pain, ST elevations and/or T wave inversions, subsequent elevation of cardiac biomarkers, and a marked decrease in LV systolic function. It is thought to be secondary to stress-induced catecholamine resurgence causing myocardial damage by microvascular spasm or direct cardiotoxicity [7]. Its diagnosis is based on Mayo Clinic’s criteria, defined as transient LV dysfunction extending beyond a single vascular territory, and all symptoms and ventricular dysfunction must occur in the absence of recent significant obstructive coronary artery disease (CAD), myocarditis, hypertrophic cardiomyopathy, or pheochromocytoma [3,6]. Cancer and administration of chemotherapy have been described among many triggers to cause TCM [8]. Cancer and chemotherapy are commonly accompanied by increased psychological stress and elevated sympathetic nervous tone, therefore, increasing the risk of TCM [9]. We report a case of TCM and associated LV thrombus as a complication of 5-FU chemotherapy that was treated with apixaban and aspirin.

## Case presentation

A 42-year-old Caucasian woman with a recent diagnosis of anorectal cancer presented to tertiary care hospital with chest pain, shortness of breath, and palpitations. She started having intermittent chest pain and heaviness three days prior to presentation. Her chest pain was typical for stable angina, i.e., localized to the central chest, worsened with activity, and improved with rest. Her review of systems was significant for cough productive of orange-colored sputum, palpitations, lightheadedness, fatigue, blurred vision, oral sores, and hallucinations. She denied fevers, chills, orthopnea, paroxysmal nocturnal dyspnea, or lower extremity edema. She was started on 5-FU chemotherapy five days ago for her cancer and denied any anginal symptoms prior to chemotherapy. Her oncological history was significant for biopsy and imaging proven stage IIa squamous cell carcinoma of the anus. She underwent radiotherapy a week prior to admission and was started on five days of continuous 5-FU infusion. The patient had a remote history of smoking one pack per day, but otherwise she had no history of diabetes or hypertension, or a family history of accelerated CAD.

On presentation, the patient’s vital signs were notable for temperature of 36.4°C, blood pressure of 104/83 mm Hg, heart rate of 133 beats per minute, respiratory rate of 27 per minute, and oxygen saturation of 94% on 3 liters of oxygen via nasal cannula. Physical examination revealed dry oral mucosa with supple neck, no jugular venous distention, and carotid bruit. Cardiovascular examination revealed tachycardic rate with regular rhythm and no murmurs, rubs, or gallops. Pulmonary examination was significant for crackles from the bases to the mid-lung fields bilaterally. Extremities showed no clubbing, cyanosis, and edema, and pedal pulses were symmetrically intact.

Investigations

Laboratory investigations at the time of presentation are illustrated in Table 1.

**Table 1:** Patient's laboratory results, particularly elevated troponins BNP, B-type natriuretic peptide

Test	Results	Normal range
Serum creatinine	0.86 mg/dL	0.6-1.10 mg/dL
Serum sodium	132 mmol/L	135-145 mmol/L
Serum potassium	4.5 mmol/L	3.5-5.1 mmol/L
BNP	3650 pg/mL	<100 pg/mL
White blood cell count	18.6 K/L	4.0-11 K/uL
Absolute neutrophil count	17.5 K/L	1.1-6.1 K/uL
Hemoglobin	12.0 gm/dL	12.0-16.0 gm/dL
Platelet count	153 K/uL	140-440 K/uL
Lactic acid	2.5 mmol/L	0.7-2.5 mmol/L
Procalcitonin	0.22 ng/mL	0.5 ng/mL
Serum troponin (first)	29 ng/mL	<14 ng/mL
Serum troponin (second)	54 ng/mL	<14 ng/mL
Prothrombin time	18.8 s	10.3-13.8 s

**Figure 1:**
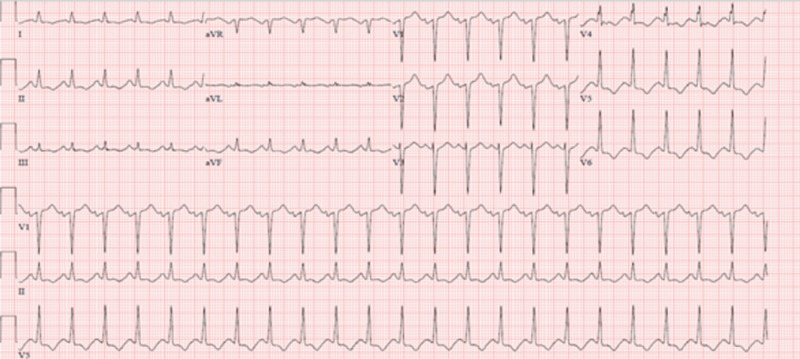
Electrocardiogram (EKG) demonstrates normal sinus rhythm with ST elevation in anterior leads and ST depression in inferolateral leads

Electrocardiogram (EKG) on presentation showed normal sinus rhythm with a regular rate of 136 beats/minute and ST changes including ST elevation in anterior leads and ST depression in inferolateral leads (Figure 1).

**Figure 2:**
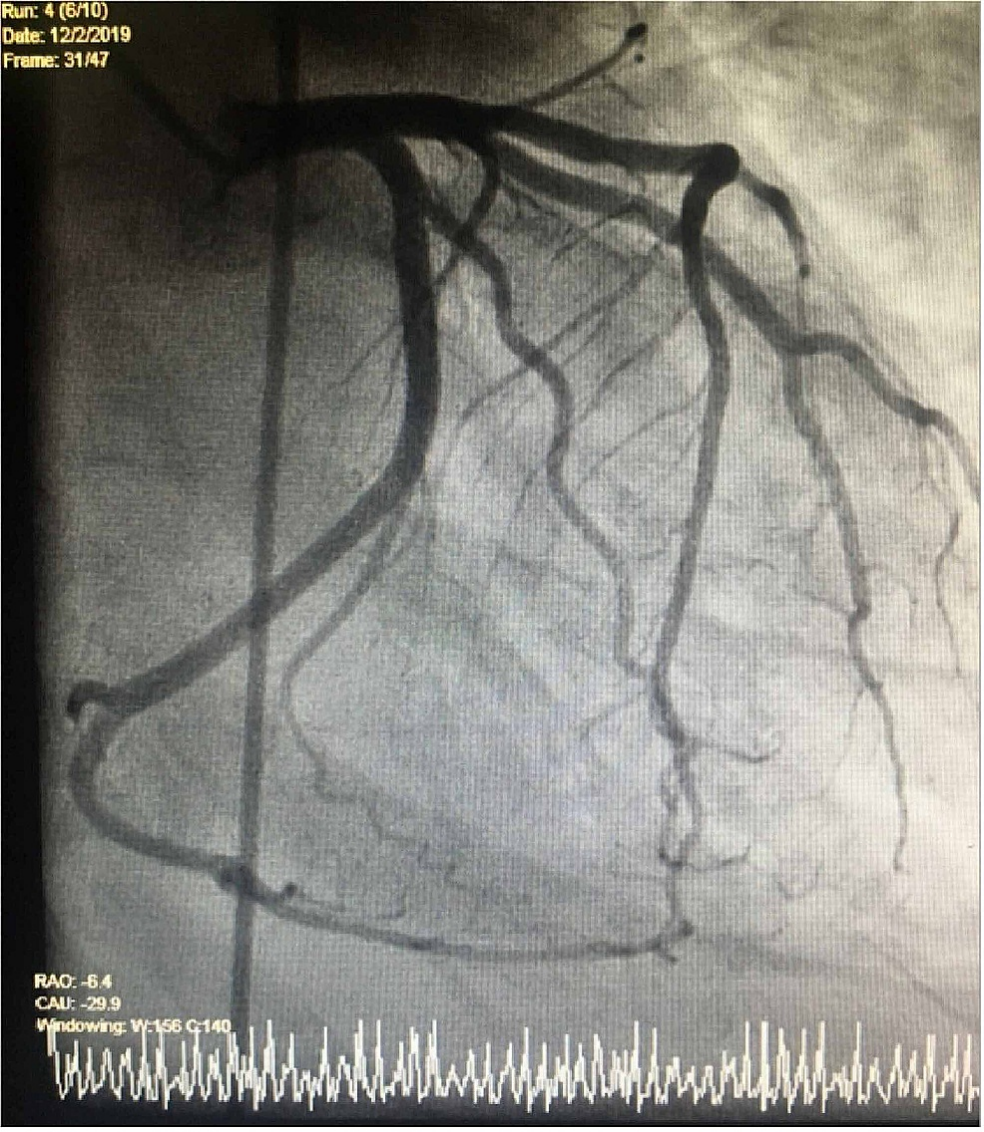
Cardiac angiogram shows a non-occlusive left anterior descending artery

**Figure 3:**
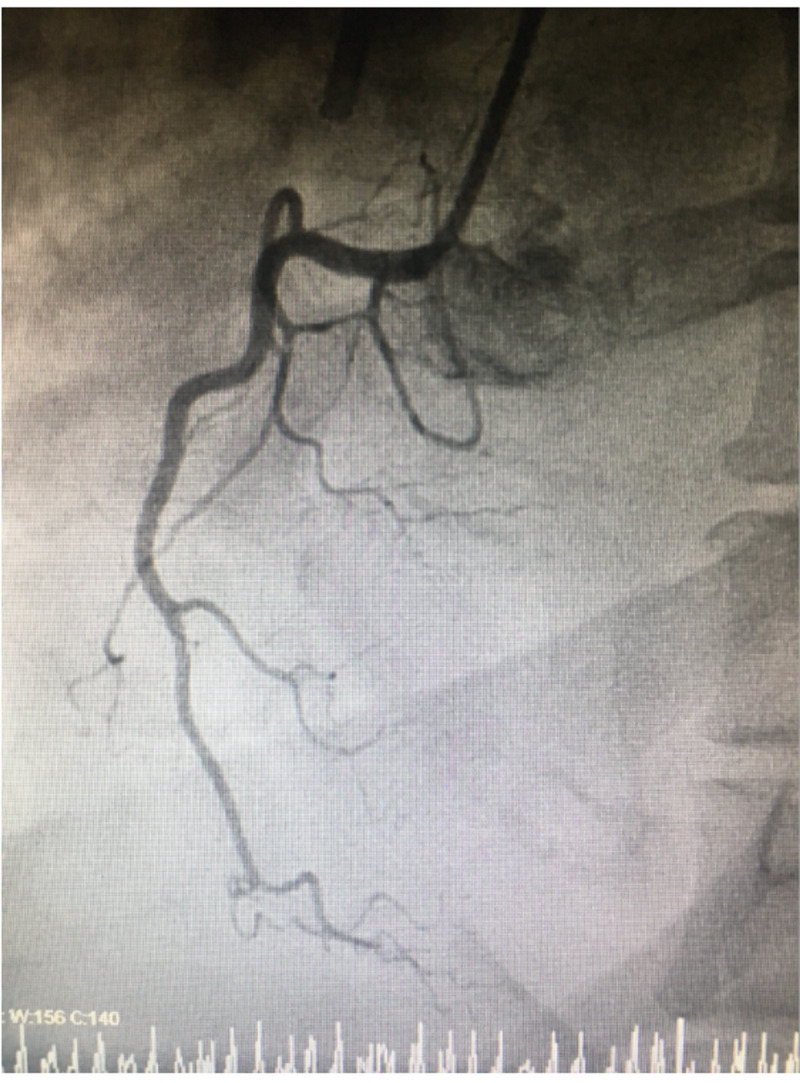
Cardiac angiogram shows a non-occlusive right coronary artery

Infectious workup showed negative respiratory infectious disease panel, no growth for five days on blood cultures, and normal flora on respiratory culture. Computed tomographic (CT) angiography of the chest showed no pulmonary embolism but revealed bilateral pleural effusions and diffuse bilateral ground-glass opacities, raising suspicion for pneumonia. Transthoracic echocardiogram (TTE) showed severely reduced LV systolic function with an estimated ejection fraction of 17%. It also showed severe global hypokinesis with apical akinesis and matted thrombus at the apex. The patient subsequently underwent a left heart catheterization (LHC), which showed 20% occlusion of the left anterior descending (LAD) artery, and other vessels were free of any occlusive disease (Figures 2, 3).

Differential diagnosis

The patient’s anginal symptoms were highly suspicious for acute coronary syndrome (ACS). This was supported by the EKG and cardiac biomarker findings. Diffuse ST-segment changes in the EKG suggested involvement of multiple coronary arteries. Since TTE findings were indicative of TCM, LHC was performed, which ruled out ACS. Other causes of chest pain, such as pulmonary embolism and aortic dissection, were ruled out with CT angiography of the chest. CT angiography initially showed concerns for pneumonia with lung infiltrates, but as culture data and laboratory findings resulted, her empiric antibiotics were discontinued. Acute pericarditis was ruled out based on the history of non-positional nature of the chest pain and no echocardiographic evidence of pericardial effusion. Multiple triggers were considered for TCM, but it was concluded that her condition is secondary to 5-FU because clinical manifestation of TCM started at the end of 5-FU chemotherapy and resolved completely with supportive care and discontinuation of medication.

Treatment

The patient received aspirin 325 mg, clopidogrel 75 mg, and high-dose statin at the time of admission. She was started on heparin infusion, scheduled intravenous furosemide for cardiogenic pulmonary edema, and nitroglycerin infusion due to persistent angina. Uridine triacetate 10 mg for 20 doses was started as an antidote per oncology recommendations because of concerns for 5-FU induced cardiotoxicity. After ACS was ruled out with LHC, heparin infusion was stopped and was transitioned to apixaban for LV thrombus.

At the time of admission, the patient also received azithromycin and ceftriaxone empirically for community-acquired pneumonia, but these were discontinued after respiratory and bacterial cultures came back negative, and procalcitonin was reported normal. She was discharged home on uridine triacetate 10 milligram (mg), aspirin 81 mg, apixaban 5 mg, lisinopril 2.5 mg, metoprolol 25 mg twice daily, and furosemide 40 mg twice daily.

Outcome and follow-up

She was closely followed as an outpatient in the heart failure clinic. Scheduled furosemide was switched to as needed after improvement in heart failure symptoms. Lisinopril was slowly titrated up to 10 mg per day. TTE was repeated after six weeks of hospital discharge, which showed resolution of heart failure with ejection fraction of 70% and complete resolution of LV thrombus. Aspirin and apixaban were continued for a total of three months. Metoprolol was progressively lowered in dosage and eventually discontinued.

## Discussion

Even though cardiac toxicity is a rare adverse effect, fluoropyrimidines such as 5-FU are the second most common cause of chemotherapy-induced cardiotoxicity [10]. Clinical cardiac toxicities associated with 5-FU covers a wide range of manifestations: coronary vasospasms leading to angina, myocardial infarction, dysrhythmia, cardiomyopathy including TCM, sinoatrial and atrioventricular nodal dysfunction, QT prolongation with torsade’s de pointes ventricular tachycardia, cardiac arrest, and sudden death have been reported in the literature [1,2]. In our case, the patient developed TCM after receiving continuous infusion of 5-FU chemotherapy for five days.

TCM as a result of exposure to chemotherapeutic agents is a rare but increasing phenomenon. Several anti-neoplastic agents including capecitabine, combretastatin, rituximab, vascular endothelial growth factors inhibitors, other angiogenesis inhibitors, and taxols have been implicated to be associated with TCM [9]. TCM more often affects postmenopausal women (82% to 100%), with the mean age being 62 to 75 years, but our patient presented at the age of 44 years [6]. Desai et al. [9] reported that TCM related to 5-FU had an incidence that was similar in both sexes. Proposed risk factors for 5-FU induced TCM include older age, concurrent administration of other cardiotoxic medications, and a history of cardiac disease and/or cardiovascular risk factors including hypertension, hyperlipidemia, and smoking [2,7]. Our patient had no history of underlying cardiovascular disease, but she had a remote history of smoking.

The dose and route of drug administration may play a role in the risk and severity of the cardiotoxicity. Continuous infusions and higher doses of 5-FU have been associated with a higher incidence of cardiotoxicity, and most patient’s present within the first two days of the initiation of the therapy [1,2]. Although vasospasm is a theorized mechanism for TCM that occurs with 5-FU, other potential mechanisms have been proposed [11]. In one case of 5-FU-related TCM reported by Basselin et al. [12], a negative methylergometrine test ruled out coronary spasm as the etiology for cardiomyopathy.

Furthermore, our patient developed LV thrombus, which is a well-known complication of TCM and is most likely related to transient apical asynergy combined with catecholamine-induced platelet and coagulation cascade activation [13,14]. The role of anticoagulation therapy to treat TCM-induced LV thrombus has not yet been defined. However, some reports mention that short-term anticoagulation therapy with heparin or warfarin for several weeks resolved LV thrombus [13,15,16]. Our case is unique in a way that our patient was managed with apixaban and aspirin with good outcome rather than typical mentioned therapy with warfarin or heparin. TCM generally has an excellent prognosis; greater than 90% of patients recover completely usually within four to eight weeks but sometimes as long as one year [14,16]. It is associated with 2% mortality and has a recurrence rate of up to 10% [14,17]. Repeat TTE in our patient at six weeks showed resolution of thrombosis and complete improvement in her ejection fraction.

Lastly, it is still in debate whether or not 5-FU be restarted in patients who recovered from 5-FU induced cardiomyopathy. Successful re-challenge with 5-FU has been accomplished; however, a mortality rate of 13% has been seen with re-challenge [18]. In a literature review, 18 out of 20 patients who underwent retreatment with 5-FU following cardiac side effects experienced further complications, including three myocardial infarction and two deaths [19]. Thus, the balance of evidence appears to argue against the reintroduction of 5-FU after recovery of cardiac function; however, a comprehensive risk-benefit assessment, extensive discussion with the patient, and multidisciplinary team approach is of paramount importance [19].

## Conclusions

5-FU is rarely associated with cardiovascular toxicity, including TCM. Discontinuation of the 5-FU is the paramount step in the management of the cardiotoxicity associated with 5-FU. Early recognition of this complication can prevent complications of LV thrombus and subsequent embolization. This case also highlights a successful treatment of LV thrombus with apixaban and aspirin.
